# Oropharyngeal leech infestation as an unusual cause of severe anemia and hypovolemic shock in a 2-year-old child: A case report from Ethiopia

**DOI:** 10.1016/j.idcr.2026.e02785

**Published:** 2026-09-29

**Authors:** Desalegn Kefale Semachew

**Affiliations:** Enat General Hospital, Amhara Region, Ethiopia

**Keywords:** Leech infestation, Oropharyngeal, Epistaxis, Severe anemia, Hypovolemic shock, Pediatrics, Ethiopia

## Abstract

**Background:**

Leech infestations can cause significant, often unrecognized, blood loss. This type of infestation occurs most commonly in young children living in rural settings who consume unfiltered water from rivers.

**Case presentation:**

A two-year-old female patient presented to a general hospital from a rural area of Ethiopia with a five-day history of epistaxis, blood-stained saliva, fatigue, and poor appetite. On examination, hemoglobin was 3 g/dL. A dark brown, mobile foreign body — subsequently identified as a leech — was found attached to the posterior oropharynx. The patient (weight 11 kg) received 20 mL/kg of intravenous fluids for resuscitation and two sequential 20 mL/kg whole-blood transfusions (cumulative 40 mL/kg) for anemia. Once stabilized, the leech was removed by direct laryngoscopy using McGill forceps. Hemoglobin had risen to 11 g/dL by the sixth hospital day, and the patient was discharged in good condition.

**Conclusion:**

Oropharyngeal leech infestation should be considered in the differential diagnosis of sudden-onset epistaxis, blood-stained saliva, and severe anemia in children from rural settings who drink unfiltered river water. Early detection and removal of the leech can be lifesaving.

## Key learning points

• Oropharyngeal leech infestation should be included in the differential diagnosis for a pediatric patient presenting with epistaxis, salivary bleeding, and/or a history of drinking river water or other unfiltered water.

• Leech saliva contains hirudin, a highly effective anticoagulant. Its thrombin-inhibiting effect can persist for approximately 7 days after the leech is removed, during which the attachment site may continue to bleed intermittently.

• The preferred method for removing an oropharyngeal leech is direct laryngoscopy under general anesthesia with sevoflurane, using McGill or blunt-jaw forceps.

• Community-level WASH interventions — water filtration, boiling of drinking water, and safe water infrastructure — are key to preventing infestation in endemic rural areas.

## Background

Leeches are parasitic organisms belonging to the phylum Annelida, class Hirudinea [1]. They are hermaphroditic and possess two suckers: an anterior sucker containing teeth/jaws used for feeding, and a posterior sucker that provides traction for attachment to the host.

Terrestrial leeches pierce the skin directly, whereas aquatic leeches typically attach to mucous membranes and are particularly dangerous when acquired through ingestion of contaminated water [2,3].

The pathology of leech attachment is driven primarily by the pharmacologic properties of its saliva, which contains hirudin — the most potent naturally occurring anticoagulant known — together with hyaluronidase (a spreading factor that facilitates tissue penetration), local anesthetic compounds, and histamine-releasing vasodilators that increase blood flow to the bite site [4]. A single adult leech can ingest 5–15 mL of blood, roughly ten times its own body weight, in under 30 min, and bleeding at the attachment site may persist for hours to days after detachment because of continued hirudin activity [5,6].

Because young children have a proportionally limited total circulating blood volume, even a single leech can cause catastrophic blood loss and severe anemia.

Infestation typically occurs through drinking, bathing, or playing in leech-infested water [7,8]. When a leech attaches to the oropharyngeal or nasopharyngeal mucosa, it feeds silently: the local anesthetic in its saliva produces little to no pain, which commonly delays recognition and allows prolonged, unrecognized blood loss before presentation [4].

Pediatric leech infestation has previously been reported from Ethiopia and neighboring countries in the region, including nasopharyngeal, laryngopharyngeal, and pharyngeal presentations in children of varying ages [2,5,6,9,10]. This report adds to that literature by describing a toddler with severe anemia (hemoglobin 3 g/dL) and overt hypovolemic shock, a degree of hemodynamic compromise that is uncommonly documented in this age group, and discusses the diagnostic reasoning, resuscitation and airway management, and public health implications relevant to this rare but potentially life-threatening condition.

This report describes the clinical presentation, diagnostic evaluation, and management of a 2-year-old Ethiopian girl with severe anemia and hypovolemic shock due to oropharyngeal leech infestation, presumed to have been acquired from unfiltered river water used for drinking.

## Case presentation

The patient, a 2-year-old girl, presented to the pediatric emergency department of a general hospital in a rural zone of the Amhara Region, Ethiopia. Her parents reported a 5-day history of epistaxis, frequent spitting of blood-stained saliva, progressive fatigue, and anorexia. There was no prior history of similar illness, no recent travel, no use of nonsteroidal anti-inflammatory drugs (NSAIDs), and no family history of a bleeding disorder. Her immunizations were up to date for her age. Her parents reported that the family's drinking water was obtained from a nearby river.

On examination, she was acutely ill and lethargic, with a body weight of 11 kg. Vital signs were: pulse 140/minute (weak), respiratory rate 50/minute, and axillary temperature 37.0°C (98.6 °F). HEENT examination revealed pale, paper-white conjunctivae with anicteric sclerae and active blood-stained nasal discharge. The lips were dry and cracked, and pooled blood was noted in the oropharynx. Direct visualization with a tongue depressor revealed clotted blood and a dark brown, mobile foreign body attached to the posterior oropharyngeal wall. Peripheral examination showed marked palmar pallor, capillary refill time greater than 3 s, and cool extremities. Neurologic examination showed lethargy with preserved response to stimulation.

Laboratory studies showed hemoglobin of 3 g/dL, blood group B positive, no hemoparasites on peripheral smear, and random blood glucose of 8 mmol/L; other complete blood count parameters were within normal limits. The clinical diagnosis was severe anemia with hypovolemic shock secondary to acute blood loss from oropharyngeal leech infestation.

Resuscitation was initiated promptly with an intravenous bolus of 20 mL/kg normal saline, which improved perfusion. Cross-matched whole blood, 20 mL/kg, was then transfused over approximately 3 h. Because hemoglobin remained low, a second 20 mL/kg whole-blood transfusion was given over the subsequent days of admission, for a cumulative transfusion volume of 40 mL/kg. Furosemide 1 mg/kg IV was administered before and after each transfusion — that is, after initial fluid resuscitation had already restored adequate perfusion — as a precaution against transfusion-associated circulatory overload, a recognized risk of rapid whole-blood transfusion in young children; the patient was monitored closely throughout and showed no clinical signs of fluid overload.

The child was then taken to the operating room and underwent direct laryngoscopy under sevoflurane–oxygen inhalational anesthesia. A live, dark brown leech was identified on the oropharyngeal wall and removed in its entirety, base included, using McGill forceps (Fig. 1); no further bleeding occurred from the attachment site after removal.

**Fig. 1:**
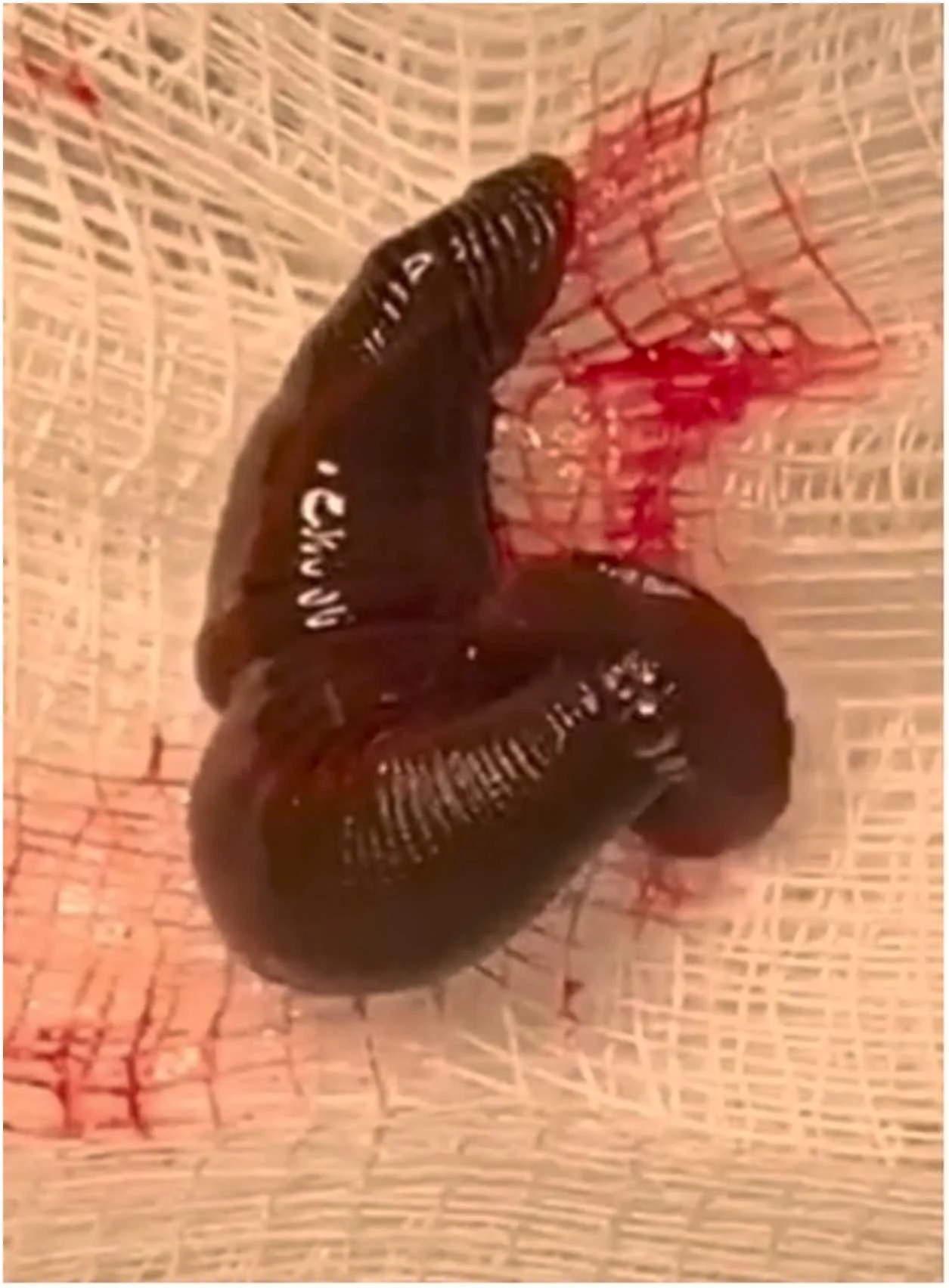
Photograph of the dark brown leech removed from the oropharynx via direct laryngoscopy and McGill forceps.

The child was transferred to the pediatric ward for ongoing observation. Over the following days she improved clinically, with no further bleeding episodes and gradual return to baseline physiologic parameters. On hospital day six, hemoglobin was 11 g/dL, and she was discharged home in good health. Before discharge, her parents were counseled that all drinking water should be boiled before consumption to prevent recurrent leech infestation.

## Discussion

This case is among a small number of documented pediatric cases in which a solitary leech caused bleeding severe enough to produce hypovolemic shock. The degree of anemia observed here (hemoglobin 3 g/dL) together with clinical evidence of hypovolemic shock is uncommon for leech infestation and, to our knowledge, represents one of the more severe hemodynamic presentations reported in a young child from Ethiopia.

The clinical manifestations of oropharyngeal leech infestation vary with attachment site. Nasopharyngeal infestation typically causes epistaxis and nasal obstruction. Laryngeal or tracheal attachment can produce hoarseness, hemoptysis, and respiratory distress [11,12]. Gastrointestinal infestation may present with hematemesis or melena, and vaginal infestation with abnormal bleeding [9,13].

This patient's presentation — epistaxis and blood-stained saliva from an oropharyngeal leech — is consistent with prior reports. Tabook and Tawab described a child with a nasopharyngeal leech presenting with similar bleeding [9]. Kibreab reported vaginal leech infestation causing abnormal bleeding in three adult women in Eritrea [13]. Kruger et al. described a 15-year-old Tanzanian girl who expelled a leech after days of hemoptysis and pallor [6], and Agin et al. reported severe anemia from a pharyngeal leech in a Turkish child [5]. Within Ethiopia, Shitaye and Shibabaw reported a 6-year-old boy with hematemesis and laryngopharyngeal leech infestation managed with transfusion and laryngoscopic removal [10]. Compared with these reports, the present case is distinguished by the patient's very young age, the severity of anemia, and the presence of overt hypovolemic shock, a combination that underscores how quickly a single leech can produce life-threatening hemorrhage in a toddler given the proportionally small circulating blood volume at this age.

A range of differential diagnoses must be systematically excluded in a toddler presenting with oropharyngeal bleeding and severe anemia. Portal hypertension with esophageal varices would typically be accompanied by signs of chronic liver disease, such as hepatosplenomegaly or ascites, which were absent here. Peptic ulcer disease and gastritis are more common in critically ill patients or those taking NSAIDs, neither of which applied to this patient. Mallory-Weiss tears are usually preceded by forceful non-bloody vomiting, which was not reported. Esophageal foreign bodies typically cause choking and drooling rather than anemia of this magnitude. Bleeding disorders, including immune thrombocytopenic purpura, hemophilia, and hematologic malignancy, must also be considered in any child with significant hemorrhage; the absence of a family history of bleeding, petechiae or bruising, and prior bleeding episodes made these less likely in this case, although formal coagulation studies and a platelet count were not performed and would have strengthened this exclusion — this is acknowledged as a limitation of the case workup.

Leech infestation should remain high on the differential for children from rural communities who rely on river water for drinking, particularly when oropharyngeal bleeding, progressive pallor, and severe anemia are present.

Hirudin, the thrombin inhibitor in leech saliva, remains pharmacologically active for a period after the leech detaches, which explains why leech wounds continue to bleed for days after the leech has left the site. Hyaluronidase breaks down surrounding tissue, extending the area the leech can penetrate, while vasodilatory compounds in the saliva keep the attachment site well perfused, and a local anesthetic delays detection and pain response during feeding.

Complete removal of the leech is essential: rupturing the leech or leaving a fragment in place allows continued hirudin secretion into the tissue and ongoing local anticoagulation and bleeding. Several removal techniques have been described; direct laryngoscopy under general anesthesia is preferred, and sevoflurane is favored because it allows the leech to release its hold before removal, reducing the risk that it will break apart. McGill or blunt-jaw forceps are most commonly used, and topical lidocaine applied to the anterior sucker, when available, can help reduce resistance to removal.

A single 20 mL/kg whole-blood transfusion would typically be expected to raise hemoglobin by approximately 2–3 g/dL, which alone would not account for the rise from 3 g/dL to 11 g/dL observed here. This 11 kg child, however, received two sequential 20 mL/kg whole-blood transfusions during the admission (cumulative 40 mL/kg), which, together with six days of ongoing hematopoietic recovery, is more consistent with the magnitude of improvement recorded by hospital day six.

## Conclusion

Oropharyngeal leech infestation is a rare but potentially lethal condition that should be considered among the causes of epistaxis, blood-stained saliva, and severe anemia, particularly in children from rural areas who rely on unfiltered river water. Prompt recognition, appropriate resuscitation, and laryngoscopic removal are central to treatment. Community-level health education on safe drinking-water practices, together with public investment in safe water infrastructure — including water filtration and treatment systems, promotion of boiling drinking water in endemic rural areas, and broader Water, Sanitation, and Hygiene (WASH) interventions — represent the most effective strategies for preventing this condition.

## Abbreviations

CNS: central nervous system; g/dL: grams per deciliter; HEENT: head, eyes, ears, nose, and throat; ITP: immune thrombocytopenic purpura; MD: medical doctor; NSAIDs: nonsteroidal anti-inflammatory drugs; OPD: outpatient department; OR: operating room; PT: prothrombin time; aPTT: activated partial thromboplastin time; RBS: random blood glucose; TACO: transfusion-associated circulatory overload; WASH: Water, Sanitation, and Hygiene.

## CRediT authorship contribution statement

**Desalegn Kefale Semachew:** Writing – review & editing, Writing – original draft.

## Ethics approval and consent to participate

Ethics approval for this case report was obtained from the Enat General Hospital Ethical Committee, under the Amhara Regional Health Bureau, prior to initiation of this work. The institutional clearance letter is available upon request from the corresponding author.

## Ethical Clearance

Ethical clearance for this case report was obtained from the Enat General Hospital Ethical Committee, under the Amhara Regional Health Bureau, prior to initiation of this work. The institutional clearance letter is available upon request from the corresponding author.

## Consent for Publication

Written informed consent was obtained from the patient's father for the publication of this case report and any accompanying images.

## Author Contributions

Dr. Desalegn Kefale Semachew was the attending physician, conceived and drafted the manuscript, and coordinated the patient's overall clinical management. Dr. Gebremikael G/wold, MD, was involved in the direct laryngoscopic removal of the leech. Mr. Animut Gashaye (Anesthetist) administered and managed the sevoflurane–oxygen inhalational anesthesia during the procedure. Mr. Moges Mihretu (Nurse) provided bedside nursing care, including monitoring during resuscitation and blood transfusion. All authors read and approved the final manuscript.

## Funding

This case report received no specific grant or funding from any agency in the public, commercial, or not-for-profit sectors.

## Declaration of Competing Interest

The author declares that he has no known competing financial interests or personal relationships that could have appeared to influence the work reported in this paper.

## Data Availability

All data supporting the findings of this case report are included within the article. Further inquiries can be directed to the corresponding author.
